# Intravesical ichthyosis: a rare case report

**DOI:** 10.1186/s12894-021-00939-9

**Published:** 2021-12-17

**Authors:** T. Hermans, R. grosse Siemer, F. C. von Rundstedt

**Affiliations:** Department of Urology, Helios University Clinic Wuppertal, Heusnerstrasse 40, 42283 Wuppertal, Germany

**Keywords:** Ichthyosis vesicae, Intravesical condylomata accuminata, Case report

## Abstract

**Background:**

Ichthyosis is a rare skin disorder, in which the shedding of squamous cells is altered. Intravesical ichthyosis is an extremely rare condition. There is evidence for an association with intravesical condylomata accuminata, caused by urogenital infections of the human papilloma virus. These lesions are generally benign but known to be of a carcinogenic potential and therefore should be treated immediately and followed-up closely.

**Case presentation:**

We present the case of a 39-year-old woman who presented with recurrent urinary tract infections. During cystoscopy diffuse black pigmented flat bladder tumours were visualized. After transurethral resection the pathological report diagnosed an ichthyosis vesicae.

**Conclusion:**

We recommend a complete resection with frequent clinical and cystoscopic follow-up. Furthermore, testing for the human papilloma virus should be performed and a vaccination should be offered to the patient. As ichthyosis vesicae is a rare phenomenon, there is an evident lack of clinical data regarding therapy, prognosis and follow-up. With our report, we want to emphasize the need for further research.

## Background

Ichthyosis is a rare inherited or acquired benign skin disease, in which the shedding of squamous cells is altered. This leads to thick dry skin patches that are made of layers of dead skin cells [1]. The acquired variant is most often associated with cancer, kidney failure, certain types of medication and hypothyroidism [2].

Intravesical ichthyosis is defined by an extensive squamous metaplasia of the bladder surface epithelium with the formation of hyperkeratotic cell deposits, but without signs of malignancy.

The phenomenon is extremely rare, with only a few case reports on PubMed (e.g. Heinzmann et al. [3]). There appears to be a causal link to intravesical condylomata accuminata (CA), most commonly known as benign genital warts and caused by the human papilloma virus (HPV) [3–6]. It has been suggested that intravesical condylomata accuminata might be precancerous regarding a further progress to squamous cell carcinoma (SCC) [3, 6, 7]. Reports of intravesical CA are very rare as well, with less than 25 cases described in literature. We know that HPV-viruses can cause cervical and several other urogenital SCCs [8]. Especially genotypes 16 and 18 are known for their malignant potency.

Very little is known about causality, treatment options and prognosis of intravesical ichthyosis.

## Case presentation

A 39-year old woman with a history of irritative LUTS with macrohematuria and recurrent proven urinary infections (4–5 per year) over more than 10 years was directed to us by her attending urologist. During cystoscopy diffuse atypical flat black pigmented bladder tumors were seen throughout the bladder. An initial tentative diagnosis was melanosis of the bladder [4].

**Fig. 1:**
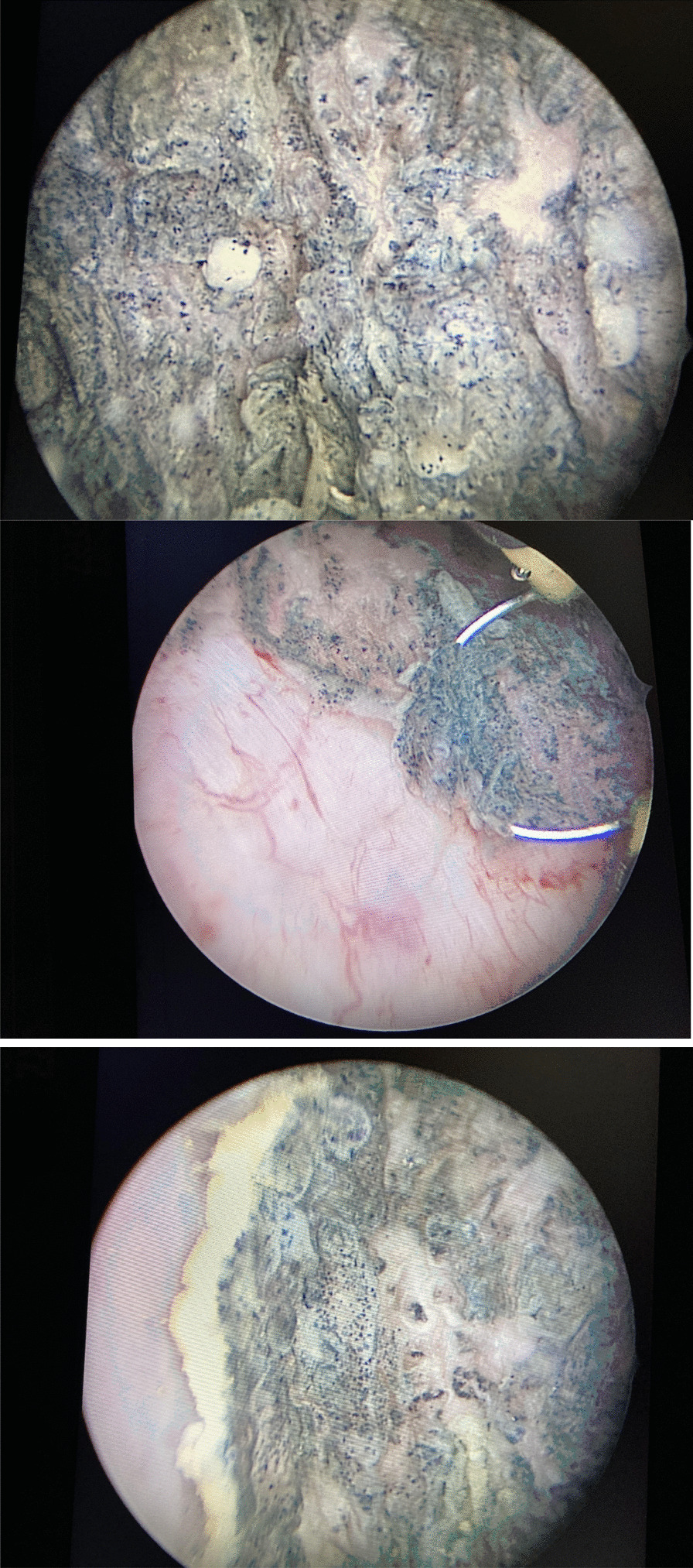
Black pigmented superficial lesions are seen ubiquitarily throughout the bladder. They penetrate the submucosa, which is seen here upon cystoscopy and during TUR-B

**Fig. 2:**
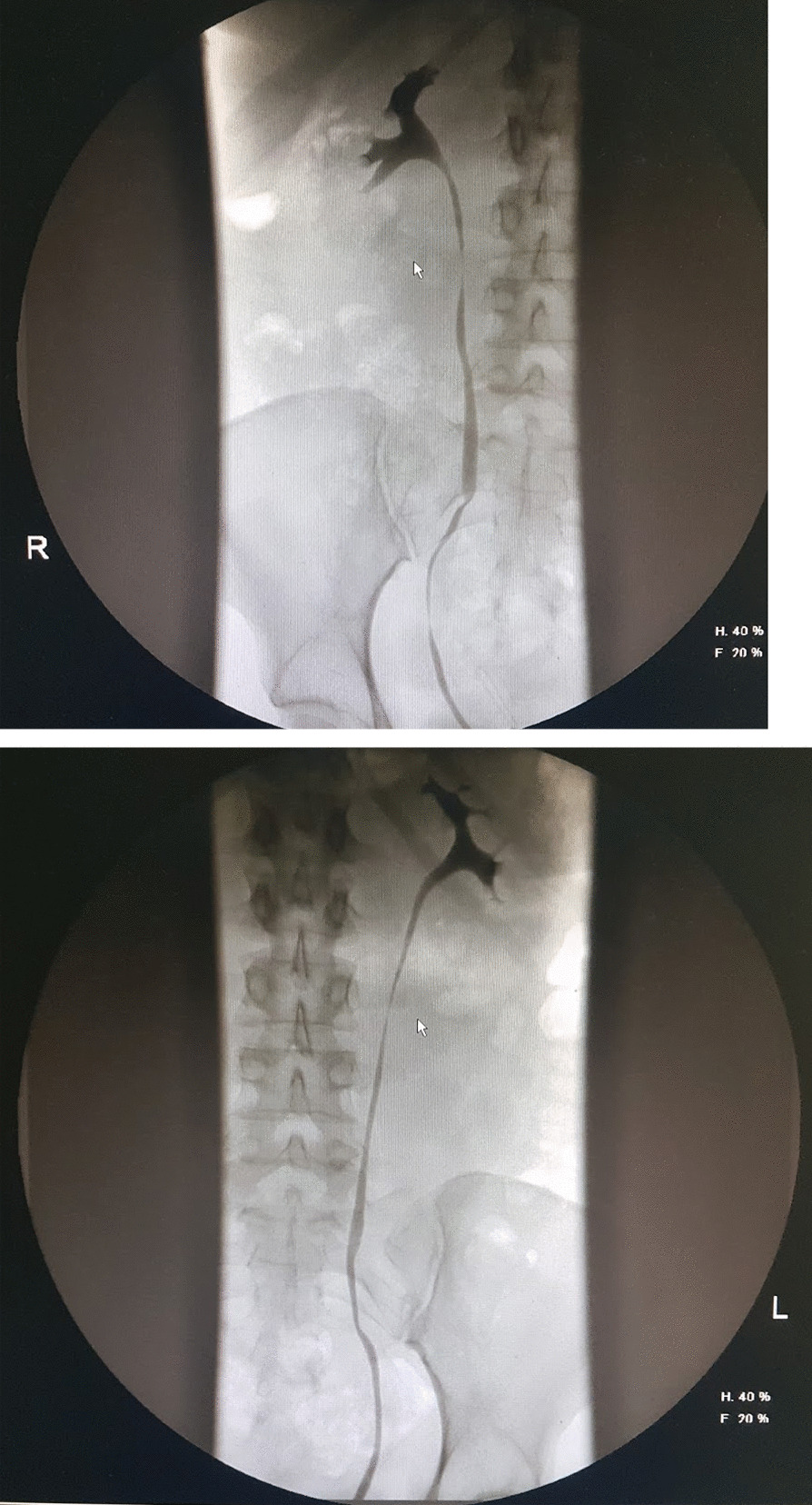
The upper urothelial tract was normal, as seen in this bilateral retrograde ureteropyelogram

The patient received an extensive transurethral bladder resection (TUR-B), in which most (but not all) of the tumor formations were resected. In multiple locations, a thick layer of black pigmented cells was scraped away from a healthy underlying urothelial submucosa (Fig. 1). Retrograde ureteropyelography showed no evidence of intraureteral lesions (Fig. 2).

**Fig. 3:**
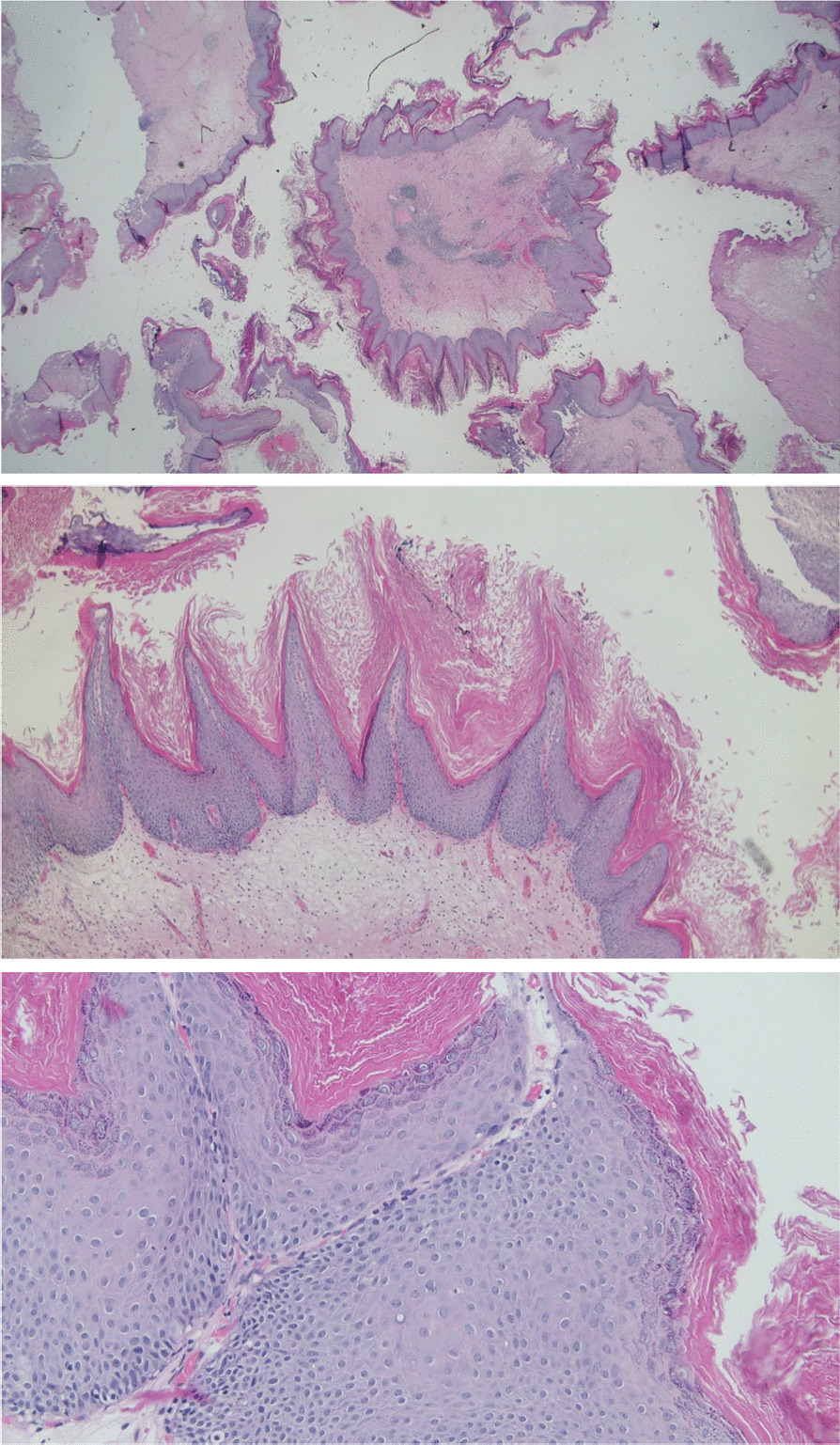
Ichthyosis vesicae. Diffuse keratinizing squamous metaplasia was seen in nearly all biopsies (25 ×, HE) with orthokeratosis, papillomatosis, hypergranulosis of the squamous epithelium and partly vacuolated keratinocystes (koilocytes) but without nuclear atypia (50 × & 200 ×, HE)

Histologically, condyloma-like benign hyperkeratotic squamous cell deposits could be seen in all resection samples (Fig. 3) and the diagnosis of intravesical ichthyosis was made. Urine cytology showed no signs of malignancy. The preoperative urine culture only showed natural skin flora (100 CFU/ml).

A profound anamnesis revealed that there was a 2-time history of extravesical CA (cervical, 10 years ago and perineal, 3 years ago) with cystoscopically no intravesical lesions at that time. The patient’s partner had no history of condylomata and both were never vaccinated against HPV. The patients only comorbidities were hypothyroidism (treated with L-Thyroxin 75 µg daily) and a penicillin-allergy. She was in a good physical condition and had never smoked.

HPV-Screening (urethral swab) was mildly positive for HPV42-DNA, a standard HPV-Vaccine (Gardasil-9®) followed. Colonoscopy showed no signs of intestinal condylomata; one small sigmoidal tubulovillous adenoma was resected.

Postoperatively, the recurrent urinary infections with macrohematuria persisted. Two control-cystoscopies after 2 and 5 months postoperatively showed minimal persistence of ichthyosis and extensive scarring, with no signs of active growth. The next cystoscopy is planned in 3 months, with a re-TUR-B if lesions progress.

## Discussion and conclusions

Condylomata accuminata are seldom found to grow intravesically. When they do, they tend to present with recurrent urinary tract infections and flat papillary lesions on cystoscopy. In most reported cases of bladder ichthyosis, and in our case as well, there has been a history of extra- (and intra)vesical CA. There is also a remarkable histological similarity between both entities, with benign pigmented hyperkeratotic squamous cell deposits being typical for both. Therefore, we hypothesize that ichthyosis vesicae is an exaggerated clinical presentation of intravesical CA. As literature on this topic is sparse (there are only 5 documented cases), further research is needed to examine this new hypothesis.

The danger of this disease seems to be a progression into muscle-invasive SCC as described by Heinzman et al. [3]. In her case report a patient with ichthyosis vesicae, who initially was under cystoscopic follow-up, developed a pT3 muscle-invasive SCC and had to undergo a cystectomy.

Besides intravesical ichthyosis, in the urogenital area only a few cases of intrauterine ichthyosis have been documented. There appears to be a causal link between ichthyosis uteri and the development of SCC as well [9–11]. O. Fadare describes a case in which a pre-existing ichthyosis uteri might have been colonized by HPV, thereby causing a spread of cervical SCC into the adjacent endometrium [9]. Other localisations of ichthyosis have not been associated with either HPV, CA or SCC.

Because of this supposed carcinogenic potential, we recommend a close clinical and cystoscopic follow-up, with re-evaluation and a re-TUR-B after 3 months. The upper urinary tract should be screened with bilateral retrograde ureteropyelography or CT urography, especially when hydronephrosis is present. If suspect, a diagnostic ureterorenoscopy can be performed to identify and treat a colonisation of the upper urinary tract. Gynaecological and colorectal screening should be included in patient workup to rule out CA early at other localisations. Furthermore, HPV-testing and -vaccination might be useful against ichthyosis vesicae. Khambati et al. [7] describe prevention of progression from CA to SCC over a period of 5 years by Gardasil vaccination and regular transurethral resections.

We conclude that intravesical ichthyosis is an extremely rare and unknown disease and we hypothesize that it is associated with intravesical condylomata accuminata. With this case report, we want to emphasize that ichthyosis should be considered as a precancerous condition in the bladder. Nevertheless, there needs to be more basic and clinical research to prove this fact.

## Data Availability

Data sharing is not applicable to this article as no datasets were generated or analysed during the current study.

## Abbreviations

CA: Condylomata accuminata
HPV: Human papillomavirus
SCC: Squamous cell carcinoma
Fig.: Figure
e.g.: Exempli gratia
al.: Alii
LUTS: Lower urinary tract symptoms
TUR-B: Transurethral resection of the bladder
CFU: Colony-forming units
DNA: Deoxyribonucleic acid
CT: Computer tomography
